# Diagnostic oriented discrimination of different Shiga toxins via PCA-assisted SERS-based plasmonic metasurface

**DOI:** 10.1515/nanoph-2024-0696

**Published:** 2025-04-17

**Authors:** Massimo Rippa, Alessia Milano, Valentina Marchesano, Domenico Sagnelli, Bryan Guilcapi, Amalia D’Avino, Giovanna Palermo, Giuseppe Strangi, Luciano Consagra, Maurizio Brigotti, Stefano Morabito, Joseph Zyss, Lucia Petti

**Affiliations:** Institute of Applied Sciences and Intelligent Systems “E. Caianiello” CNR, Pozzuoli, Italy; Department of Physics, NLHT-Lab, University of Calabria, Arcavacata, Italy; CNR-NANOTEC, Institute of Nanotechnology, 87036, Rende, Italy; Department of Physics, Case Western Reserve University, Cleveland, OH, 44106, USA; Dipartimento di Scienze Mediche e Chirurgiche, Sede di Patologia Generale, Università di Bologna, Bologna, Italy; Department of Food Safety, Nutrition and Veterinary Public Health, Istituto Superiore di Sanitá, Rome, Italy; Lumière, Matière et Interfaces (LUMIN) Laboratory, Institut D’Alembert, Ecole Normale Supérieure Paris-Saclay, Université Paris Saclay, Gif sur Yvette, France

**Keywords:** metasurface, Shiga toxin, SERS, plasmonic, PCA

## Abstract

Plasmonic biosensors are powerful platforms for detecting various types of analytes. Specifically, surface-enhanced Raman spectroscopy (SERS) can enable label-free and selective detection. Shiga toxin-producing *Escherichia coli* (STEC) represents zoonotic pathogens that cause severe diseases, such as hemolytic uremic syndrome (HUS), the most important cause of acute renal failure in children. To date, there are no effective therapies for STEC infection, and the available diagnostic methods are complex and inconclusive. Here, a novel nanopattern fabricated by electron beam lithography with remarkable plasmonic properties is employed as SERS substrate for realizing the specific recognition of Stx1a, Stx2a, and of a third variation of the latter. A limit of detection (LOD) of 6.8 pM for Stx1a and 2 pM for Stx2a was achieved. Our approach supported using the principal component analysis (PCA) appears to be a valid alternative to conventional methods, allowing real-time and fast *in situ* analysis.

## 1 Introduction

Plasmonic nanostructures have garnered significant attention in the scientific community due to their unique optical properties and potential applications in various fields, particularly in biosensing [1], [2], [3], [4], [5], [6], [7], [8], [9], [10], [11], [12], [13]. These structures, typically composed of noble metals like gold and silver, exhibit localized, propagative, or mixed surface plasmon resonances (LSPRs), which are sustained by copropagating by a hybrid electron–photon wave at the interface between metal and air or any other dielectric coverage. Due to their mixed nature, plasmons are not constrained by diffraction limits and can thus be confined to subwavelength volumes. The resulting giant enhancement of the plasmonic electromagnetic fields near the surface or singularities of these nanostructures forms the basis for their application in sensing technologies [14], [15], [16], [17], [18], [19], [20]. The concept of using plasmonic nanostructures for sensing purposes dates back several decades, with early studies demonstrating the sensitivity of LSPRs to changes in the metal dielectric interface and hence to the corresponding local refractive index. Whereas the effect was discovered in the early seventies by the adsorption of small aromatic molecules on roughened metallic surfaces fabricated by electrochemical techniques [21], advancements in nanofabrication techniques have enabled the precise control of nanostructure size, shape, and composition, further enhancing their sensing capabilities. Various types of plasmonic nanostructures, including nanoparticles, nanorods, and nanoshells, have been developed and employed as biosensors [22], [23], [24], [25], [26], [27], [28], [29], [30], [31], [32], [33], [34]. In the context of biosensing, plasmonic nanostructures are particularly effective due to their ability to interact with biological molecules at the nanoscale. This interaction results in measurable changes in the optical properties of the nanostructures, providing a means to detect, quantify, and eventually identify the presence of specific analytes. Their high sensitivity, specificity, and potential for miniaturization make plasmonic nanostructures ideal candidates for biosensor applications. Among the various plasmonic sensing platforms, surface-enhanced Raman spectroscopy (SERS) is a powerful analytical technique that combines the molecular specificity of Raman spectroscopy with the signal enhancement provided by plasmonic nanostructures [35], [36], [37], [38], [39], [40], [41]. When molecules are adsorbed onto the surface of plasmonic nanostructures, the Raman scattering cross section is significantly enhanced, often by several orders of magnitude. This enhancement allows for the detection of low-abundance biomolecules that would otherwise be undetectable using conventional Raman spectroscopy. The mechanism behind SERS involves two primary enhancement effects: electromagnetic enhancement and chemical enhancement. Electromagnetic enhancement is attributed to the amplification of the local electromagnetic field generated by the LSPRs of the nanostructures [42], [43], [44]. Chemical enhancement, on the other hand, involves charge transfer between the adsorbed molecules and the metal surface, leading to an increase in Raman scattering intensity [44]. Additionally, plasmonic nanostructures can be engineered with nanoscale features so as to extend the one-dimensional vertical confinement from flat metallic surfaces to three dimensional volumes, favoring in particular lateral confinement, enhancing the signal and allowing for the miniaturization of the diagnostic device. SERS has been successfully applied in various bioanalytical applications, including the detection of nucleic acids, proteins, and small molecules [45], [46], [47], [48], [49], [50], [51]. The technique offers several advantages, such as high sensitivity, multiplexing capability, and the ability to perform label-free detection. Recent advancements in SERS have focused on the development of novel plasmonic substrates, improved reproducibility, and the integration with microfluidic systems for point-of-care diagnostics of various types of targets. Among the analytes of interest, Shiga toxins, produced by pathogenic *Escherichia coli* strains (*E. coli*) and *Shigella dysenteriae*, are potent cytotoxins that play a critical role in the pathogenesis of several intestinal diseases [52], [53], [54], [55], [56], [57], [58], [59], [60]. These toxins are classified into two main types: Shiga toxin 1 (Stx1) and Shiga toxin 2 (Stx2), each with several subtypes (e.g., Stx1a, Stx2a, Stx2c, and Stx2d). Structurally, Stxs consists of an enzymatically active A subunit and a pentameric B subunit responsible for binding to host cell receptors. The primary mechanism of action of Stxs involves the inhibition of protein synthesis in target cells. The B subunit binds to globotriaosylceramide (Gb3) receptors on the surface of host cells, facilitating the entry of the A subunit into the cytoplasm. Once inside the cell, the A subunit cleaves a specific adenine residue from the 28S ribosomal RNA, halting protein synthesis and leading to cell death. Shiga toxins are major contributors to the development of hemolytic uremic syndrome (HUS), a severe condition characterized by acute renal failure, hemolytic anemia, and thrombocytopenia [61]. HUS primarily affects young children and the elderly and can lead to significant morbidity and mortality. It is a life-threatening condition that often arises as a complication of intestinal infections with Shiga toxin-producing *E. coli* (STEC). The pathophysiology of HUS involves the dissemination of Shiga toxins in blood, their binding to circulating cells and the subsequent release by leukocytes and platelets of toxin-containing extracellular vesicles that target endothelial cells of the kidney leading to platelet activation, and the formation of microthrombi. The resulting microangiopathic hemolytic anemia, thrombocytopenia, and acute renal impairment are the hallmarks of HUS. Recently, it has been found that Stx2a circulates in the blood of STEC-infected children in two different forms, which differ by only a single cleavage in the A subunit. Cleaved Stx2a A chain is split into two fragments (A1 and A2) held by a disulfide bond. Native noncleaved Stx2a and cleaved Stx2a have different binding properties for human circulating cells thus showing different contributions to the pathogenesis of HUS [62]. Epidemiologically, HUS is most seen in children under the age of five, though it can occur to individuals of any age. Moreover, STEC that produces Stx2 are epidemiologically associated with HUS, whereas the risk of developing the syndrome is very low in patients infected by STEC producing Stx1 and approximately halves when infecting bacteria produce both toxins [63]. Outbreaks of STEC-associated HUS have been reported worldwide, often linked to the consumption of contaminated food or water. The long-term health impacts of HUS can be severe, including chronic kidney disease, hypertension, and neurological complications. The acute phase of HUS requires prompt medical intervention to manage the symptoms and prevent further complications. This includes supportive care such as fluid management, blood transfusions, and dialysis. However, the effectiveness of treatment is heavily dependent on the rapid and accurate diagnosis of the condition. The diagnosis of STEC infections and HUS currently relies on a combination of clinical presentation, laboratory tests, and microbiological assays. Traditional methods, such as culture-based techniques and immunoassays, can be time-consuming and may lack the sensitivity required for early detection. Rapid and *in situ* diagnosis is crucial for the timely administration of appropriate therapies and for preventing the progression of the disease. Point-of-care diagnostic tools that can provide accurate and immediate results are, therefore, highly desirable. Sensors based on plasmonic effects may be suitable for this purpose. However, the use of these types of sensors for the detection of Stx is rarely found in literature. Nagatsuka et al. [64] and Yaghoubi et al. [65] exploited the LSPR property of gold nanoparticles to detect Stx. Zhang et al. [66] developed a surface plasmon coupling electrochemiluminescence assay based on gold nanoparticles for the determination of the Stx gene. Wang et al. [67] proved that surface plasmon resonance imaging immunosensors can achieve a rapid and label-free microarray detection of Stx1 and Stx2. There are even fewer works in literature regarding the use of the SERS technique. SERS-based sensors can offer high sensitivity and specificity for the detection of Stxs, enabling their detection at very low concentrations. Yang et al. [68] developed a SERS sensor based on Ag nanostructure to rapidly detect a target ss-DNA (*stx2*). Ren et al. [69] made use of a colorimetric and SERS dual mode aptasensor based on gold nanostars loaded with metalorganic framework for the specific detection of the Stx2. Finally, our group [70] as case of study of a novel plasmonic pattern used as SERS substrate, achieved the specific detection of the Stx2a with a limit of detection (LoD) of 1.4 nM. In the present paper, a planar plasmonic metasurface was employed for SERS analysis to achieve both the fingerprint spectrum and the specific recognition of Stx1a, Stx2a, and cleaved Stx2a. The metasurface used was composed of an ordered hexagonal array of nanoelements fabricated by means of electron beam lithography (EBL), recently studied and characterized showing extremely interesting optical properties such as second-harmonic generation. In particular, we demonstrated that the pattern considered can exhibit an intrinsic superchiral response, comparable with the results obtained for out-of-plane 3D structures, although our sample is characterized by a very thin thickness (<*λ*/10). Such distinctive properties are related to the special shape of the designed inclusions that lead to a strong enhancement of the near field in the visible and NIR spectral range, due to the presence of a significant density of hot-spots that characterizes both the distribution of the electric and magnetic fields [71], [72]. The acquired SERS spectra were analyzed using the principal component analysis (PCA), which can further improve analytical capabilities, enabling the differentiation and classification of different Stxs by enhancing the subtle differences between their unique spectral signatures. Our results demonstrate how the proposed method can potentially represent a valid alternative to the conventional ones for the detection of Stxs and their classification allowing a label free real-time analysis. Moreover, the detection system designed can be integrated with other biological on-chip devices to realize portable point-of-care medical diagnostics.

## 2 Materials and methods

### 2.1 Metasurface fabrication and morphological characterization

**Figure 1:**
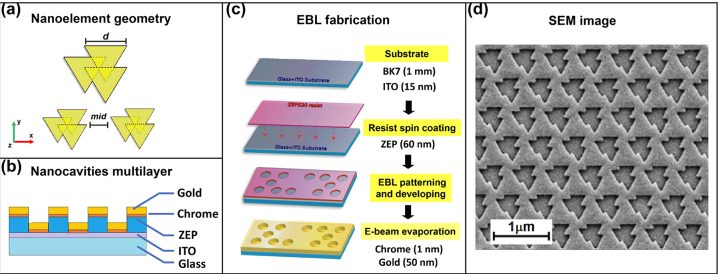
Plasmonic metasurface fabricated. (a) Schematic representation of the nanoelement geometry. (b) Nanocavities multilayer fabricated. (c) The main steps of the EBL fabrication process employed. (d) SEM image of the final plasmonic metasurface realized.

The 450 μm × 430 μm gold meta-surface patterns follow an ordered hexagonal array made of individual nanoelements abiding to trigonal symmetry. Such nanostructures, referred to as octupolar entities in view of their absence of dipolar contributions to their noncentrosymmetric structures, had been conceived and developed toward second-harmonic generation, an important nonlinear optical phenomenon per se and for characterization of surfaces, while providing a relevant testbed toward further biosensing applications [26], [27], [28], [29] fabricated by using the EBL system Raith 150. Nanoelements consist of 3 equilaterals triangular NCs with side *d* of 200 nm partially overlapped (Figure 1a). In the process, a 150 nm layer of positive resist (styrene methyl acrylate, ZEP 520A) was spin coated on a 15 nm conductive ITO coated glass substrate, baked at 170° for 5 min, and exposed to a 10.2 pA electron beam with an area dose of 30 *μ*C/cm^2^. The NCs were achieved in the resist film after development in an n-amyl acetate solvent, then rinsed for 90 s in 1:3 MIBK:IPA solution for 60 s, followed by IPA rinse for 30 s. Finally, the gold array was obtained by e-beam evaporation (SISTEC CL-400C) of 2 nm of chrome and 50 nm of gold film onto the resist layer. The schema of the obtained multilayer is shown in Figure 1b, while the main steps of the fabrication process are represented in Figure 1c. The minimum interparticle distance (*mid*) in the *x* direction was 100 nm. The nanostructures fabricated were characterized morphologically by making use of scanning electron microscopy (SEM, Raith 150). A SEM image of one of the nanopattern realized is shown in Figure 1d.

### 2.2 Toxins and antibodies

Stx1a and Stx2a produced by *E. coli* C600 (H19J or 933W, respectively) were purified by receptor analog affinity chromatography on globotriose–fractogel (IsoSep, AB) (Ryd, M. et al., (1989) FEBS Lett. 258, 320–322) or on (Gal1-4 Gal -O-spacer)-BSA Sepharose 4B (Glycorex, Lund, Sweden) [73], respectively. Both toxins were passed through ActiCleanEtox columns (SterogeneBioseparations, Carlsbad, CA, USA) to remove endotoxin contaminant. Cleaved Stx2a [62] was obtained by treating native Stx2a (120 µg) with 1 µg of trypsin in 100 µL phosphate-buffered saline (PBS) pH 7.5 (1 h at 37 °C) followed by the addition of 20 µL of 0.7 μg/ml trypsin inhibitor phenylmethylsulfonyl fluoride (10 min at 37 °C). Purified toxins were stored at −80 °C in small aliquots and diluted before each assay with PBS. Monoclonal antibodies (Toxin Technology, Sarasota, Florida, United States) to Stx1 (Stx1–13C4) and Stx2 (Stx2-BB12) were used for the functionalization of the proposed nanostructures.

### 2.3 Metasurface functionalization and Shiga toxin deposition

We realized the proposed biosensor using a noncovalent functionalization of the nanopatterned gold surface through physisorption of the monoclonal antibodies for Stxs (AbStx1 for Stx1a and AbStx2 for STx2a and Stx2a cleaved). In detail, 50 μl of 10 μg/mL monoclonal antibody in phosphate-buffered saline (PBS) was deposited dropwise onto the nanostructured chip surface, and the solution was incubated in a humid chamber overnight at 4 °C to achieve antibody physical absorption on the gold surface. After that, the chip was washed 5 times with 1 mL of bi-distilled water to remove the unfixed antibody. To reduce the aspecific signal, we adopted a blocking strategy before toxin deposition onto the biofunctionalized nanostructures. We used 1 % w/w BSA to passivate the sensor surface (1h at 25 °C in a humid chamber). Then, the chip was copiously washed again with bi-distilled water. Right after, the biosensor performances were assessed using a toxin solution with a final concentration of 45 nM. The toxin solution was dropped on the functionalized nanostructures and left to incubate in a humid chamber overnight at 4 °C. After incubation of the analyte, the biosensor was washed five times with 1 mL of bi-distilled water and dried with N_2_ before SERS spectrum data collection.

### 2.4 SERS measurement: set-up and parameters

SERS spectra were recorded using a Raman system (QE Pro-Raman, Ocean Optics) coupled with an upright microscope (Olympus BX51) in a backscattering configuration. The system was configured for *λ* = 785 nm with a grating of 1,200 lines/mm and an input slit of 50 *μ*m. Spectra were collected using a 50 × (N.A. = 0.75) objective and a laser power of 12 mW. In the case of Shiga toxin sensing, for each sample, 40 SERS spectra from different points were collected in the range of 350–1,400 cm^−1^ with an acquisition time of 10 s. In all cases investigated, spectra recorded were subsequently baseline corrected and averaged using a homemade MATLAB (R2019b, Math-Works) code. For the power level used in the measurements, corresponding to a beam intensity of approximately 1.7 × 10^3^ W/cm^2^, photothermal tests were conducted to demonstrate that no significant temperature increase in the sample was observed. Details related to this issue are reported in the Supplementary Material.

### 2.5 PCA

Principal component analysis (PCA) is a statistical technique used to reduce the dimensionality of datasets while preserving the most important variance. In this study, PCA was employed to discriminate between Stx1a, Stx2a, and cleaved Stx2a, as their SERS spectra are nearly identical, making visual differentiation extremely difficult. This distinction is of great clinical importance for accurate toxin identification. The analysis was performed using R version 2024.04.2+764, with the ChemoSpec package. After the PCA, an analysis of variance (ANOVA) was applied to the first two principal components (PC1 and PC2), using the stats package in R to determine if significant differences existed between the toxin groups identified. Specifically, a one-way ANOVA was performed on each principal component independently to evaluate the effect of the different toxins on the spectral features extracted by PCA.

## 3 Results and discussion

In this work, we address the challenge of developing a SERS-based detection system that allows rapid and *in situ* analysis of Stxs, a group of powerful cytotoxins responsible for the pathogenesis of different human diseases. To pursue our aim, we used an engineered planar plasmonic metasurface that we developed, studied, and characterized in previous works [71], [72]. The selection of our engineered planar plasmonic metasurface was guided by the need to optimize SERS performances while ensuring high reproducibility, tunability, and compatibility with a wide range of applications, including biological sensing. While numerous plasmonic structures have been explored for SERS, many suffer from limitations such as i) irregular Hot-Spot Distribution, ii) limited Spectral Tunability, or iii) restricted Polarization Control.

To overcome these issues, we developed and employed a hexagonal array of asymmetric nanocavities (NCs) fabricated in a gold thin film. The NCs are substantially achieved through the fusion of three equilateral triangles, two aligned on one side and the third one centered on the opposite side, with an overall size of approximately 450 × 430 μm^2^ (Figure 1a and b). Figure 1d shows the SEM image of the fabricated plasmonic metasurface that highlights the good quality of both the shape of the individual NCs and their repeatability in the lattice. The asymmetry and periodicity of this pattern facilitate a high density of hot-spots and ensure efficient near-field enhancement in the spectral range of interest (750–850 nm), perfectly matching our laser source. Furthermore, the hexagonal arrangement naturally enhances plasmonic coupling between neighboring NCs, improving the overall SERS performances. Below, we highlight the key advantages of our structure:

a) Highly Reproducible and Uniform Hot-Spot Distribution: Unlike disordered nanostructures, our metasurface provides an ordered and periodic lattice that ensures a consistent SERS signal across the entire substrate.
b) Tailored Resonance and Spectral Matching: By adjusting the mid parameter (25–150 nm), we fine-tuned the structure to achieve maximum enhancement, something that is difficult to control in simpler nanostructures. The value of 100 nm considered here represents the one that guarantees the best matching with the wavelength of 785 nm of the laser source of our SERS system and, consequently, the greater amplification of the SERS signal [72].
c) Dual-Mode Excitation (Circularly Polarized & Unpolarized Light): The ability to efficiently excite multiple plasmonic modes enhances signal intensity and expands sensing capabilities, making our structure particularly suitable for multisensing applications.
d) Superchiral Response: We demonstrated that the pattern considered can exhibit an intrinsic superchiral response, and particularly a high near field enhancement in the 750−850 nm spectral range, due to the presence of a significant density of hot-spots that characterize both the distribution of the electric and magnetic fields [71]. This has been demonstrated by illuminating the structure with circularly polarized light. Similarly, using unpolarized light results in an overall excitation effect of all the possible modes of the structure resulting in a very high near field enhancement efficiency, both in terms of electric and magnetic fields (Supplementary Material). We point out that the formation of these hot spots is also favored by the hexagonal arrangement of the fabricated NCs.

Considering these interesting achievements, we exploited the potential of this pattern to design SERS substrates aimed at the analysis of Stxs.

To test our SERS substrate, among the main Stx subtypes, we selected Stx1a and Stx2a from the main Shiga toxin (Stx) subtypes associated with severe human disease. Stx1a, which is primarily linked to bloody diarrhea and rarely to hemolytic uremic syndrome (HUS), is the least associated with HUS. In contrast, Stx2a is the subtype most frequently implicated in HUS. Additionally, we included its recently described modified form (cleaved Stx2a). Rapid identification of these subtypes during STEC infections would enable the distinction between patients at low or high risk of developing HUS, providing clinicians with valuable information for patient management. In our study, we combine the information obtained by SERS measurements with the potential provided by the well-known PCA to analyze the samples investigated and to obtain their specific detection. The results achieved with two different approaches based on the use of unfunctionalized and functionalized SERS substrates, assisted by PCA, are shown and discussed below. We emphasize that, for mass fabrication, the throughput limitations of the EBL technique used here to fabricate the plasmonic nanopattern can be overcome by employing alternative methods to produce low-cost replicas of the developed nanopatterns, such as Nanoimprint Lithography (NIL), Soft Lithography, and Replica Molding (RM).

### 3.1 Stx analysis by unfunctionalized SERS substrates

**Figure 2:**
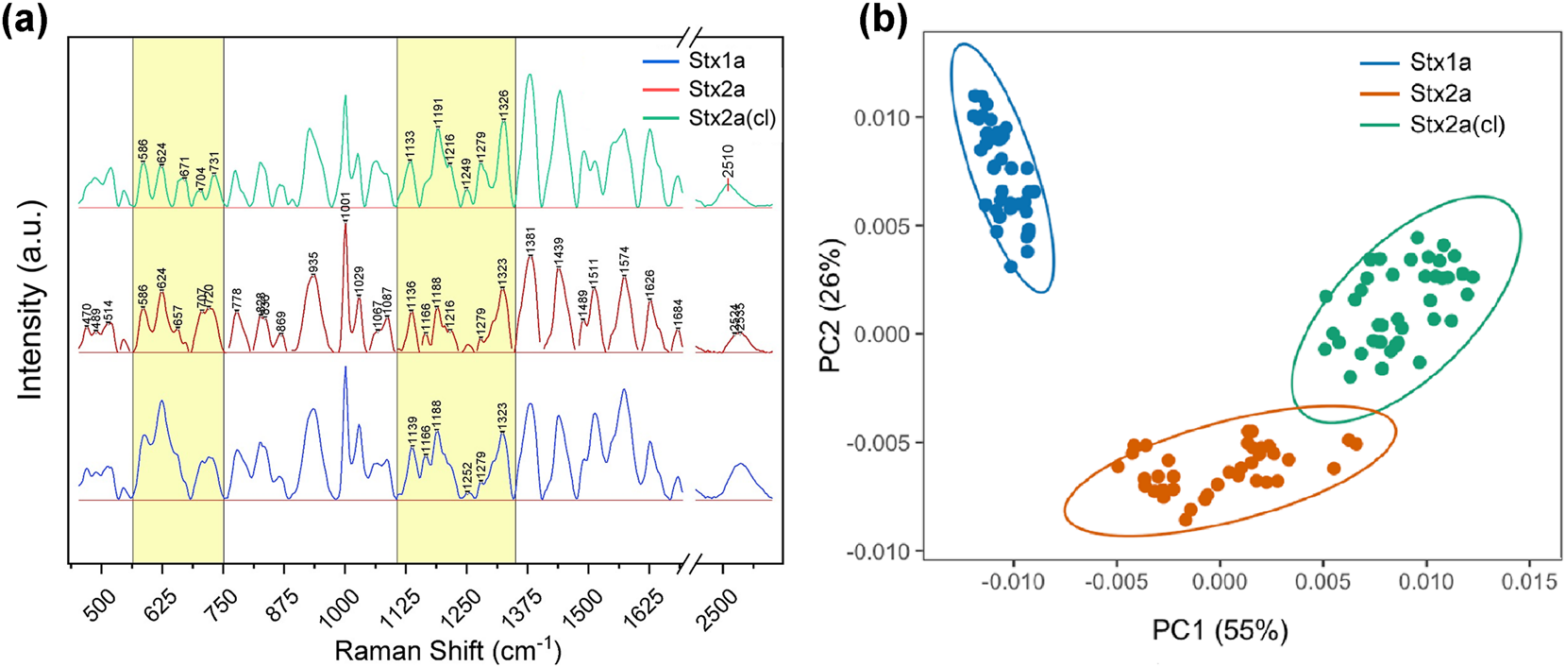
SERS analysis of the three Stx taken into consideration. (a) SERS fingerprint of the Stx1a (blue line), Stx2a (red line), and Stx2a-cl (green line). (b) PCA analysis associated with the SERS measurements of the Stx1a (blue points), Stx2a (red points), and Stx2a-cl (green points).

The plasmonic metasurface was tested to define the SERS fingerprint spectrum of the three Stx taken into consideration. The three toxins were deposited at the same concentration of 45 nM onto three equally nanopatterned metasurfaces by direct absorption. The measurements were performed on each nanopattern, which provided repeatable spectra with well-resolved peaks. Figure 2a shows the average and normalized SERS spectra obtained for Stx1a (blue line), Stx2a (red line), and its cleaved form Stx2a-cl (green line), respectively.

The fingerprint spectra shown in Figure 2a exhibits a complex and varied distribution of vibrations corresponding to the three proteins. The main spectral peaks for the three analytes are reported in Table 1. The Raman spectrum analysis identified unique peaks for the toxins (Stx1a, Stx2a, and Stx2a-cl), emphasizing their chemical fingerprints. The analysis of the spectrum of the three toxins reveals notable differences in peak shifts and intensities. The assignment table reveals numerous notable peaks, including those associated with disulfide bond stretching, tyrosine and tryptophan ring breathing modes, and backbone stretching. These peaks elucidate the structural characteristics of the toxins and their molecular interactions.

**Table 1:** Comparison and tentative assignment of the main spectral peaks found for the three analytes in their SERS fingerprint.

Wavenumber (cm^−1^)	Assignation	Stx1a	Stx2a	Stx2a(cl)
514–544	S–S stretching [70]	+	+	+
624	n(C–S)G [74] Phe [75]	+	+	+
657	C–H in-plane bending, stretch of H39 on C16, strong swagging of H42 on C24 and H28 on N2, H37 on O12, out of plane breathing of benzene ring [76]	–	+	–
671	Isoleucine/C–S stretching [70], [77]	–	–	+
720	Ile [78] met C–S stretching [70] n(C–S)T [74] Tryptophan [79]	+	+	–
836	Backbone stretching/out of plane ring breathing in tyrosine [70]	–	+	–
935	Tryptophan/ C–C stretching [79] δ(C–C–N)symm, α-helical skeletal [80], [81]			
1,001	Symmetric ring breathing mode of phenylalanine [82] Symmetric CC ring stretching [82]	+	+	+
1,029	C–H stretching mode of phenylalanine [83]	+	+	+
1,252	Amide III band -N–H in-plane bend, C–N stretch [79], [81]	+	+	+
1,279	Amide III band [79] CH2 wag or Ring stretching [82]	+	+	+
1,323	Trp/C–H deformation/backbone/C–N twisting [77] C–H stretching of adenine [83]	+	+	+
1,381	COO- stretching [79]	+	+	+
1,552	COO^−^ stretching /His/ Trp /Phe [70]	–	–	+
1,574	Amide II band - Ade/Gua [79]			
1,626	Tyr/Trp/Phe [79]	+	+	+
1,684	Amide I band [79]	+	+	+
1,723–1,836	C=O stretch and C–C stretch [84], [85]	+	–	–
2,510–2,535	S–H stretching, thiol group exposure [86]	–	–	+

The cleaved Stx2a exhibits distinct peaks (e.g., 671 cm^−1^, 731 cm^−1^, 891 cm^−1^ and 1,552 cm^−1^) and changes in thiol group exposure (2,510 and 2,524 cm^−1^), signifying substantial structural modifications following cleavage. In the spectrum of cleaved Stx2a, the C–S stretching region is dominated by the C–S bond vibration at 671 cm^−1^ but not in Stx2a and Stx1a spectra where the peak of methionine is better resolved (720 cm^−1^). Notably, cleavage of Stx2a induced the resolution of different peaks attributable to Trp (731 cm^−1^, 891 cm^−1^, and 1,552 cm^−1^) suggesting greater exposure of these aromatic amino acids compared to Stx2a. Moreover, some peaks observed in Stx2a are absent in the cleaved form (Table 1 and Table S1 in Supplementary Material), providing further evidence of the changes induced by the cleavage of Stx2a. Further differences are highlighted by two yellow boxes showing spectral differences between the cleaved and the wild type. In the 550–750 cm^−1^ band, we can see that the cleaved form shows a more definite spectra, and, in the band, comprised between 1,125 and 1,370 cm^−1^, we see again more resolved or more intense peaks. A notable example is the peak at 1,279 cm^−1^ showing how the vibration of the Amide III region corresponding to α-helix conformation (1,260−1,300 cm^−1^) is more intense, indicating again important structural differences. On the other hand, this behavior of cleaved Stx2a also demonstrates that, as in the case of the native uncleaved toxin, the protein contains a significant fraction of α-helix structure as previously shown by circular dichroism [62].

A notable distinction is in the intensities of specific peaks within the spectra. The area beneath the curve varies considerably among the different spectra. For instance, while examining the vibrations associated with S–S bonds, an increase in the area under the curve is observed between the cleaved Stx2a and wild type at 514 and 544 cm^−1^, with fold increases of 1.3 and 1.1 folds, respectively. Moreover, the cleaved isoform exhibits a change in the -SH vibrational modes relative to the wild-type. The SH bands in the cleaved form are shown by a distinct peak at 2,510 cm^−1^, whereas the wild-type exhibits modes at 2,524 and 2,535 cm^−1^. The area of the cleaved isoform SH vibrations is 1.5 times greater than that of the wild type. These data validate the alteration of the toxin structure following cleavages. Interestingly, the analysis confirms that the distinctions between Stx2a and Stx1a, originating from two different genes and thus having different primary sequence, are more likely to be significant. By comparing the spectra of the native toxins (Stx1a and Stx2a), it is clear that differences are more quantitative than qualitative since most of the peaks are present in both spectra albeit with different intensities.

Subsequently, PCA was utilized to extract key spectral information from the small differences in SERS spectra. Figure 2b shows the projected spots in the 2D space constructed by the first two principal components (PC1 and PC2), referring to the Stx1a (blue points), Stx2a (red points), and Stx2a-cl (green points) associated with the SERS measurements. As visible from the map, the points associated with the three toxins are clearly distinguishable forming three clearly separated clusters. Interestingly, the relative distances between the clusters also reflect the real differences in the chemical composition of the investigated toxins, which are very pronounced between Stx1a and Stx2a and very subtle between Stx2a and Stx2-cl (even partially overlapping).

ANOVA revealed highly significant statistical discrimination among the three toxins for both PC1 and PC2, with exceptionally low p-values (Supplementary Material). This success in separating the clusters can be explained by considering the values of the variance explained for the PC1 of 55 % and for the PC2 of 26 %, whose sum exceeds 80 %, which represents a threshold reference value to obtain an adequate diversification of the different investigated analytes.

This result demonstrates that SERS in combination with PCA can be a valuable tool for detecting differences among different samples of Stxs in water, allowing for a specific recognition of the analytes without the need for antibody probes.

### 3.2 Stx detection by functionalized SERS substrates

**Figure 3:**
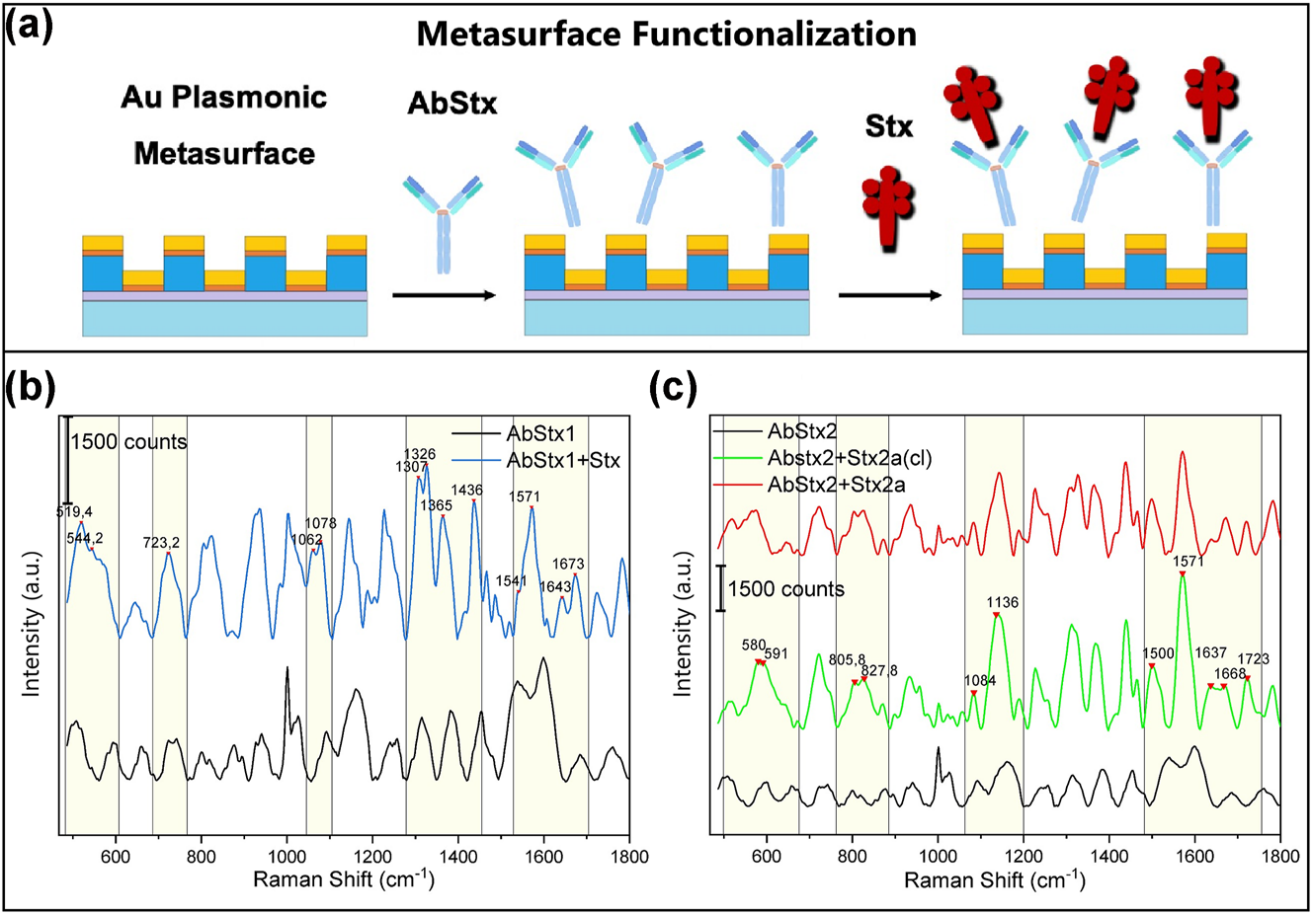
Analysis of functionalized SERS metasurface. (a) Schema of the main steps of functionalization process. (b) Comparison of the average SERS spectra measured for AbStx1 (black line) at a concentration of 10 μg/mL and AbStx1a/Stx1a (blue line). (c) Comparison of the average spectra measured for AbStx2a (black line) at a concentration of 10 μg/mL in PBS, AbStx2a/Stx2a (red line) and AbStx2a/Stx2a-cl (green line) at 154 nM in PBS.

To develop a sensor capable of detecting and discriminating the three Stxs taken into consideration, a second approach based on the use of functionalized SERS substrates was investigated. Three copies of our nanostructured substrate were functionalized with specific antibodies for Stx1a (AbStx1), Stx2a, and Stx2-cl (AbStx2) at an optimized concentration of 10 μg/mL in PBS. Subsequently, a concentration of 45 nM of the three toxins was deposited on the respective substrates as described in Section 2.2. A schema of the main steps of metasurface functionalization is shown in Figure 3a. Repeated measurements were performed on each substrate before and after the deposition of the Stxs. Figure 3b shows the comparison of the average SERS spectra obtained for AbStx1 (black line) and functionalized toxin AbStx1a/Stx1a (blue line), while Figure 3c shows a similar comparison between the average spectra achieved for AbStx2a (black line), AbStx2a/Stx2a (red line), and AbStx2a/Stx2a-cl (green line), respectively.

The comparison between the spectra of the antibody (Ab) alone and the Ab-Tox complex (Figure 3b and c) reveals significant differences, suggesting structural changes resulting from the interaction between the antibody and the toxin. Unique peaks emerge in the Ab-Tox spectra, which are absent in the Ab alone spectra. For instance, in Figure 3b, novel peaks appear in the Ab-Tox complex spectrum, such as the band between 500 and 550 cm^−1^, indicating the presence of disulfide (S–S) bonds characteristic of the toxin.

Additionally, several peaks are unique to the Ab-Tox complex. For example, the 1,574 cm^−1^ peak, also evident in the fingerprint, is attributed to amide II vibrations, highlighting changes in the protein secondary structure upon toxin binding. These distinct spectral features are also observed in complexes with both Stx2a and Stx2a(cl) antibodies. In Figure 3c, the region between 1,500 and 1,700 cm^−1^ shows peaks specific to the toxin (e.g., 1,574 cm^−1^) and to the Ab-Tox complex (e.g., 1,500 cm^−1^), further emphasizing the structural differences introduced by the interaction.

**Figure 4:**
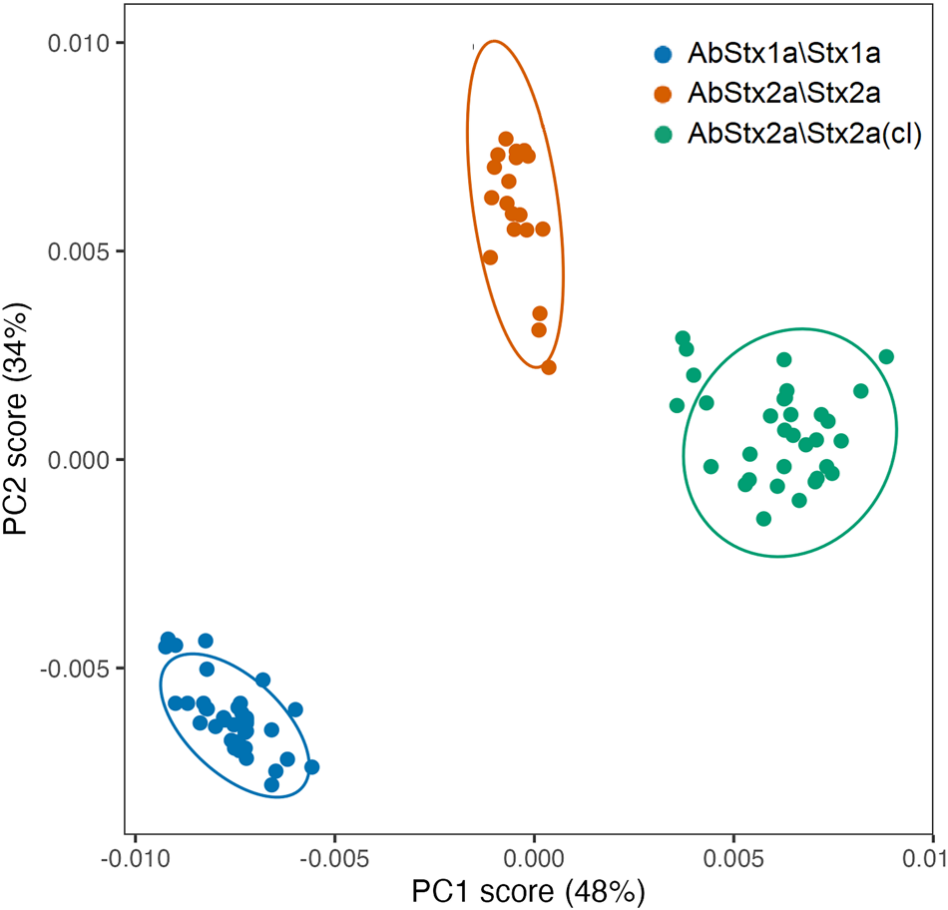
PCA analysis of the SERS spectra measured in the presence of the toxins: AbStx1a/Stx1a (blue points), AbStx2a/Stx2a (red points), and AbStx2a/Stx2a-cl (green points).

Subsequently, as applied in the previous approach, PCA was used to discriminate against the SERS signals measured in the presence of the toxins. Figure 4 shows the spots in 2D space referring to PC1 and PC2 of the SERS measurements carried out for AbStx1a/Stx1a (blue points), AbStx2a/Stx2a (red points), and AbStx2a/Stx2a-cleaved (green points).

As well visible, the points associated with the three toxins are clearly distinguishable forming three separate clusters. Interestingly, like the SERS fingerprint behavior discussed above, the relative distances between the clusters reflect the actual differences in the chemical composition of the three toxins.

The sum of the variance values explained for PC1 (48 %) and PC2 (34 %) also for this experimental condition exceeds 80 %, allowing for adequate diversification of the different analytes investigated. Similarly, in the previous case, ANOVA was performed on the measured data, revealing highly statistically significant discrimination among the three toxins for both PC1 and PC2 (Supplementary Material).

We point out that while a separation of the clusters characterizing Stx1a and the two forms of Stx2a analyzed could have been well expected due to the different and specific antibodies used, the same result was not obvious for the measurements carried out in the presence of Stx2a and Stx2a-cleaved for which the same antibody was used. This result highlights even more how the information obtained from the PCA analysis can confirm and enrich what can be obtained and deduced from the simple analysis of the spectra shown in Figure 3, allowing fine discrimination even between subspecies of toxins which are in fact chemically very similar.

Subsequently, to further test the performance in the detection of the developed sensing system, we determined the limit of detection (LoD) for both Stx1a and Stx2a. To this aim, we measured SERS spectra of the toxin in PBS at different concentrations of the toxins lower than 1 nM, after functionalizing 2 copies of the plasmonic metasurface with 10 μg/mL of the respective antibodies.

**Figure 5:**
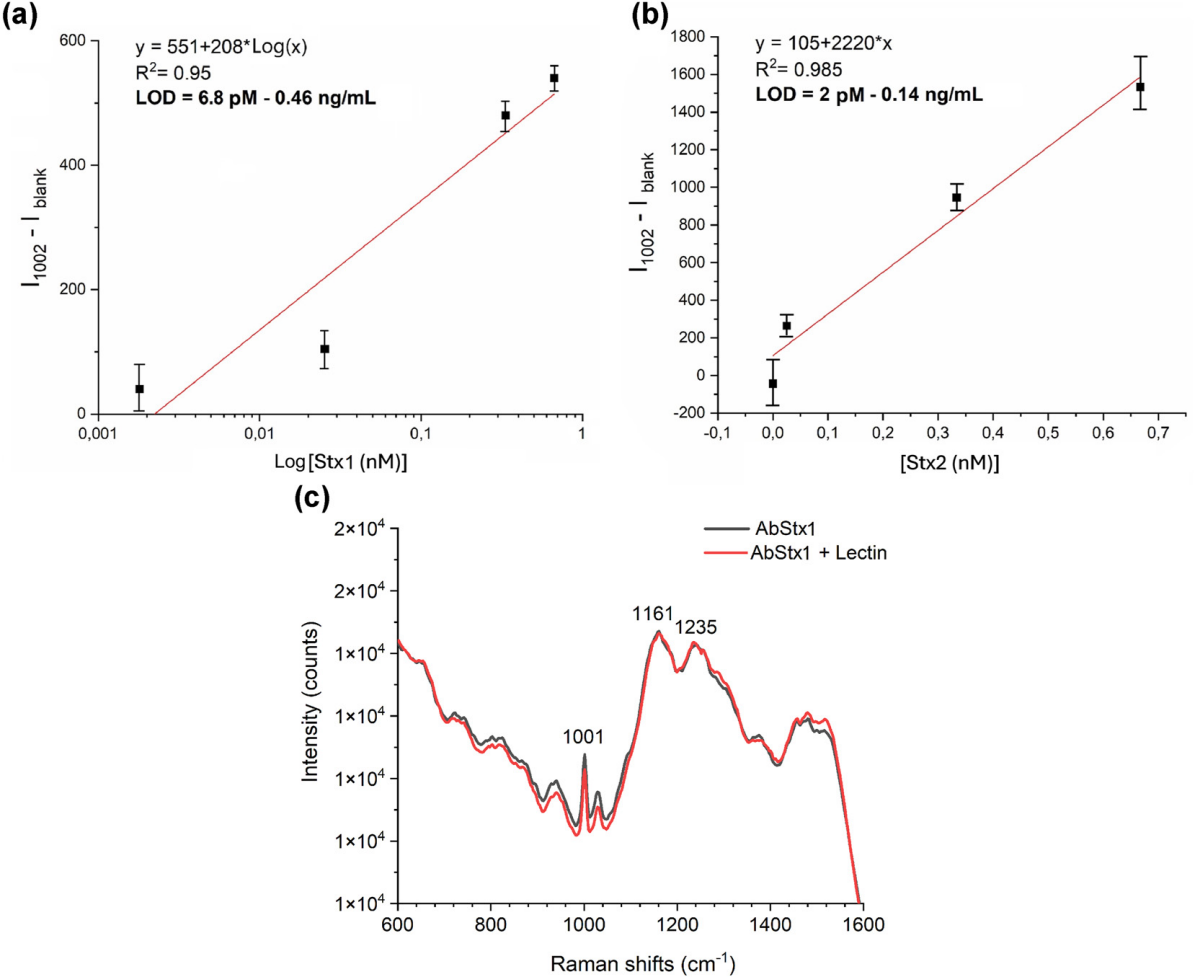
Test of functionalized SERS metasurface: a) LoD evaluation for Stx1 detection, experimental points obtained considering the intensity of the 1,002 cm^−1^ peak (black squares) and linear fit associated with data (red line); b) LoD evaluation for Stx2 detection, experimental points obtained considering the intensity of the 1,002 cm^−1^ peak (black squares) and linear fit associated with data (red line); c) specificity test, comparison, of the SERS spectra measured for the Stx1 antibody alone (black line) and after incubation of 154 nM of the Lectin toxin (red line).

In Figure 5a and b, the *y*-axis reports the values obtained subtracting the Raman intensities (*I*) of the highest peak 1,002 cm^−1^, measured in the presence of the toxin, from the intensity of the same peak (*I*_*blank*_) measured before toxin incubation (with antibody alone). In the case of the Stx1a, the experimental points show a good linear relationship between the quantity *I − I*_*blank*_ and the logarithmic values of the toxin concentration *C*. The best fit for the relationships ***I − I***_***blank***_ *= a + b* ***Log(C)*** was achieved with *a* = 551, *b* = 208, and R^2^ = 0.95 (red line in Figure 5a). Conversely, for Stx2a, a linear relationship ***I − I***_***blank***_ *= a + b* ***C*** provided the best fit, with a = 105, b = 2,220, and R^2^ = 0.985 (red line in Figure 5b).

In both cases, the LOD was determined following the standard method recommended by the International Union of Pure and Applied Chemistry (IUPAC) [87]. IUPAC defines the LOD as the lowest concentration of an analyte that can be reliably distinguished from background noise. A commonly used approach, particularly with linear calibration curves, defines the LOD as the concentration at which the signal equals the blank signal plus three times the standard deviation (3σ). Here, σ represents the standard deviation of blank measurements or low-concentration samples, reflecting the inherent variability of the analytical signal in the absence of the analyte. The factor 3 ensures statistical confidence, meaning that the detected signal exceeds background noise with a probability of approximately 99.7 %, assuming a normal distribution. This method is scientifically robust, as it is based on well-established statistical principles and aligns with the IUPAC definition of detection limits. Following this approach, the standard deviation (σ) was calculated based on multiple blank measurements. By solving the best-fit equation for this threshold, we obtained LOD values of 6.8 pM (0.46 ng/mL) for Stx1a and 2 pM (0.14 ng/mL) for Stx2a.

We observe that the LOD values estimated for our proposed system are superior or at least competitive with which of both plasmonic systems and traditional methods reported in the literature, summarized in Table 2.

**Table 2:** Plasmonic approaches [64], [65], [66], [67], [68], [69] and traditional methods [88], [89], [90], [91], [92], [93], [94], [95] reported in the literature for Shiga toxin identification.

Technique	Feature	Analyte	LOD	Ref.
LSPR platform	Synthetic glycosyl ceramides attached to gold nanoparticles	Stx	10 ng/mL	[64]
LSPR platform	Gold nanoparticles bound to antibodies	Stx2	10 ng/mL	[65]
SPC-ECL	Nanospheres of Au NPs encapsulated into a solid silica core with graphite phase carbon nitride quantum dots embedded	Stx	9 fM	[66]
SPRi	Biochip with 50-nm gold film	Stx1, Stx2	50 ng/mL	[67]
SERS substrate	Recycled silicon chips	Stx2	0.3158 μM	[68]
SERS device	Noble metal nanoparticles and Raman reporter loaded metal–organic framework	Stx2	0.82 ng/mL	[69]
LC-MS	Nanospray liquid chromatography-mass spectrometry and Vero cell bioassay	Stx	Stx2c (5.7 ng/mL)	[88]
Reverse latex agglutination	VTEC-Screen Seiken	Stx1, Stx2	25 ng/ml	[89]
Nanoparticle platform	Gold nanoparticles conjugated with Gb3 and silver enhancement	Stx1	1 μg/ml	[90]
Nanoparticle platform	Magnetic nanoparticles conjugated with Gb3 and MALDI-TOF	Stx1	330 pg/ml	[91]
(mPCR) assay	Vero cells in a three-dimensional (3D) platform	Stx1, Stx2	32 ng/ml	[92]
Lateral flow immunoassay	Based on AuNP and CdTe QD	Stx2	25 ng/ml	[93]
SERS device	2D hybrid metallic polymeric nanostructure based on the octupolar framework	Stx2	1.4 nM	[94]
AlphaLISA	Bead-based immunoassay	Stx	0.5 ng/mL	[95]

Finally, preliminary measurements were conducted to evaluate the specificity of our system for Shiga toxin detection using a nontarget toxin, specifically a lectin (Erythrohemagglutinin PHA-E (*Phaseolus vulgaris*)).

PHA-E lectin was chosen as a representative nontarget analyte because, like Shiga toxin, it is a biologically active toxin that can be found in the bloodstream in acute pathogenic syndromes. This selection aimed to assess whether our sensor could distinguish between Shiga toxin and another toxin potentially present in similar biological environments. Figure 5c shows the SERS spectra obtained for the Stx1a antibody alone (black line) and after incubation with 154 nM of the lectin in PBS (red line). Despite the extremely high concentration of the lectin compared to that used for Stx, no signal amplification was observed. The SERS spectrum achieved reflects the characteristic signal of antibody alone, with no contribution from the lectin.

These results highlight the potential of the proposed sensing system as a highly sensitive and specific label-free platform for the detection of Shiga toxins, making it an excellent candidate for diagnostic applications where precision and reliability are critical.

## 4 Conclusions

In conclusion, this work explores the sensing performance of a novel plasmonic metasurface based on a periodic arrangement of EBL-fabricated NCs that has shown promising opto-plasmonic properties in previous works. We used our system as a SERS substrate to achieve Stxs recognition. In particular, we combine the spectral information obtained from SERS measurements with the statistical analysis provided by PCA to study and detect three different Stxs: Stx1a, Stx2a, and its cleaved version Stx2-cl. The results obtained with the nonfunctionalized approach demonstrate the possibility of discriminating the three toxins through the spectral fingerprints measured and, more clearly, through the PCA analysis performed. Furthermore, the results obtained with the functionalized approach show the possibility of carrying out the detection of three toxins, allowing fine recognition even between subspecies chemically similar. The most ominous complication of STEC infections in children is HUS that develops about a week after the onset of prodomal intestinal symptoms (e.g., bloody diarrhea) when Stxs target the kidney. Therefore, the proper management of STEC-infected children depends on early diagnosis. Moreover, the discrimination between low-risk STEC (Stx1-producing), high-risk STEC (Stx2-producing), or intermediate-risk STEC (producing both Stx1 and Stx2) is of paramount importance for the institution of therapy [53], [96]. In the functionalized approach, the very low LODs of about 0.46 ng/mL and 0.14 ng/ml estimated for Stx1a and Stx2a, respectively, also highlight the ability of the developed system to carry out analysis and detection of concentrations of the investigated cytotoxins that are lower than those found in patients’ sera (2–6 ng/ml by ELISA) before the development of HUS [97]. Finally, the ability of the system to distinguish two forms of Stx2a (native and cleaved) differently involved in the pathogenesis of HUS provides an added value prompting investigations on their role in the clinical presentation of patients. These findings demonstrate that the plasmonic metasurface proposed is very promising for the development of devices for sensitive detection of the analytes considered at the point of care. Future efforts of this research activity will be directed toward testing solutions in real matrices and integrating the proposed sensing system into Lab on Chip type platforms also for point-of-care (POC) analysis.

## Supplementary Material

Supplementary Material Details

## References

[1] Altug H., Oh S. H., Maier S. A., Homola J. (2022). Advances and applications of nanophotonic biosensors. *Nature Research*.

[2] Hamza M. E., Othman M. A., Swillam M. A. (2022). Plasmonic biosensors: review. *Biology*.

[3] Jin C., Wu Z., Molinski J. H., Zhou J., Ren Y., Zhang J. X. J. (2022). Plasmonic nanosensors for point-of-care biomarker detection. *Elsevier B.V.*.

[4] Vestri A. (2021). LSPR immuno-sensing based on iso-Y nanopillars for highly sensitive and specific imidacloprid detection. *J. Mater. Chem. B*.

[5] Chen D., Zhou J., Rippa M., Petti L. (2015). Structure-dependent localized surface plasmon resonance characteristics and surface enhanced Raman scattering performances of quasi-periodic nanoarrays: measurements and analysis. *J. Appl. Phys*..

[6] Mejía-Salazar J. R., Oliveira O. N. (2018). Plasmonic biosensing. *Am. Chem. Soc.*.

[7] Rippa M., Castagna R., Tkachenko V., Zhou J., Petti L. (2017). Engineered nanopatterned substrates for high-sensitive localized surface plasmon resonance: an assay on biomacromolecules. *J. Mater. Chem. B*.

[8] Rippa M. (2018). Dodecagonal plasmonic quasicrystals for phage-based biosensing. *Nanotechnology*.

[9] Kravets V. G., Kabashin A. V., Barnes W. L., Grigorenko A. N. (2018). Plasmonic surface lattice resonances: a review of properties and applications. *Am. Chem. Soc.*.

[10] Rippa M. (2020). Octupolar plasmonic nanosensor based on ordered arrays of triangular Au nanopillars for selective rotavirus detection. *ACS Appl. Nano Mater*..

[11] Paria D., Kwok K. S., Raj P., Zheng P., Gracias D. H., Barman I. (2022). Label-free spectroscopic SARS-CoV-2 detection on versatile nanoimprinted substrates. *Nano Lett*..

[12] Lai W., Zhou J., Jia Z., Petti L., Mormile P. (2015). Ag@Au hexagonal nanorings: synthesis, mechanistic analysis and structure-dependent optical characteristics. *J. Mater. Chem. C Mater.*.

[13] Dhama R. (2018). Extraordinary effects in quasi-periodic gold nanocavities: enhanced transmission and polarization control of cavity modes. *ACS Nano*.

[14] Fang Z., Zhu X. (2013). Plasmonics in nanostructures. *Adv. Mater.*.

[15] Sonntag M. D., Klingsporn J. M., Zrimsek A. B., Sharma B., Ruvuna L. K., Van Duyne R. P. (2014). Molecular plasmonics for nanoscale spectroscopy. *Roy. Soc. Chem.*.

[16] Han J. H., Kim D., Kim J., Kim G., Fischer P., Jeong H. H. (2023). Plasmonic nanostructure engineering with shadow growth. ..

[17] Challenges and advances in computational Chemistry and physics 15. ..

[18] Kumari A., Vyas V., Kumar S. (2023). Synthesis, characterization, and applications of gold nanoparticles in development of plasmonic optical fiber-based sensors. *Inst. Phys.*.

[19] Rippa M. (2017). Octupolar metastructures for a highly sensitive, rapid and reproducible phage-based detection of bacterial pathogens by SERS. *ACS Sens.*.

[20] Bochenkov V. E., Shabatina T. I. (2018). Chiral plasmonic biosensors. *MDPI*.

[21] Mcquillan A. J. (2009). The discovery of surface-enhanced Raman scattering. *Notes Rec. R.*.

[22] Zohar N., Chuntonov L., Haran G. (2014). The simplest plasmonic molecules: metal nanoparticle dimers and trimers. *Elsevier B.V.*.

[23] Haran G., Chuntonov L. (2018). Artificial plasmonic molecules and their interaction with real molecules. *Am. Chem. Soc.*.

[24] Klimov V. V., Guzatov D. V. (2007). Plasmonic atoms and plasmonic molecules. *Appl. Phys. A Mater. Sci. Process.*.

[25] Zyss J. (1993). Molecular engineering implications of rotational invariance in quadratic nonlinear optics: from dipolar to octupolar molecules and materials. *J. Chem. Phys*..

[26] Salomon A., Zielinski M., Kolkowski R., Zyss J., Prior Y. (2013). Size and shape resonances in second harmonic generation from silver nanocavities. *J. Phys. Chem. C*.

[27] Salomon A., Prior Y., Fedoruk M., Feldmann J., Kolkowski R., Zyss J. (2014). Plasmonic coupling between metallic nanocavities. *J. Opt. (United Kingdom)*.

[28] Kolkowski R., Szeszko J., Dwir B., Kapon E., Zyss J. (2014). Effects of surface plasmon polariton-mediated interactions on second harmonic generation from assemblies of pyramidal metallic nano-cavities. *Opt. Express*.

[29] Kolkowski R., Petti L., Rippa M., Lafargue C., Zyss J. (2015). Octupolar plasmonic meta-molecules for nonlinear chiral watermarking at subwavelength scale. *ACS Photonics*.

[30] Barrow S. J. (2016). Electron energy loss spectroscopy investigation into symmetry in gold trimer and tetramer plasmonic nanoparticle structures. *ACS Nano*.

[31] Chuntonov L., Haran G. (2013). Optical activity in single-molecule surface-enhanced Raman scattering: role of symmetry. *MRS Bull*..

[32] Chuntonov L., Haran G. (2013). Maximal Raman optical activity in hybrid single molecule-plasmonic nanostructures with multiple dipolar resonances. *Nano Lett*..

[33] Yan B., Boriskina S. V., Reinhard B. M. (2011). Design and implementation of noble metal nanoparticle cluster arrays for plasmon enhanced biosensing. *J. Phys. Chem. C*.

[34] Wallace G. Q., Lagugné-Labarthet F. (2019). Advancements in fractal plasmonics: structures, optical properties, and applications. *Roy. Soc. Chem.*.

[35] Kneipp K., Moskovitis M., Kneipp H. (2019). *Surface-enhanced Raman Scattering: Physics and Applications*.

[36] Beeram R., Vepa K. R., Soma V. R. (2023). Recent trends in SERS-based plasmonic sensors for disease diagnostics, biomolecules detection, and machine learning techniques. *MDPI*.

[37] Zhou L. (2020). Irreversible accumulated SERS behavior of the molecule-linked silver and silver-doped titanium dioxide hybrid system. *Nat. Commun*..

[38] Kneipp K. (1997). Single molecule detection using surface-enhanced Raman scattering (SERS). *Phys. Rev. Lett.*.

[39] Liang Z. (2019). SERS-based cascade amplification bioassay protocol of miRNA-21 by using sandwich structure with biotin-streptavidin system. *Analyst*.

[40] Liu Y. (2015). Hydrothermal synthesis of gold polyhedral nanocrystals by varying surfactant concentration and their LSPR and SERS properties. *RSC Adv*..

[41] Rippa M. (2024). Fractal plasmonic molecule for multi-sensing: SERS platform for SARS-CoV-2 detection. *ACS Appl. Nano Mater*..

[42] Kneipp K. (2007). Surface-enhanced Raman scattering. *Phys Today*.

[43] Anker J. N., Hall W. P., Lyandres O., Shah N. C., Zhao J., Van Duyne R. P. (2008). Biosensing with plasmonic nanosensors. *Nat. Mater.*.

[44] Kambhampati P., Child C. M., Foster M. C., Campion A. (1998). On the chemical mechanism of surface enhanced Raman scattering: experiment and theory. *J. Chem. Phys*..

[45] Lin C. (2023). Recent development of surface-enhanced Raman scattering for biosensing. *J. Nanobiotechnology*.

[46] Issatayeva A. (2024). SERS-based methods for the detection of genomic biomarkers of cancer. *Talanta*.

[47] Stanfoca Casagrande G. M. (2024). SERS sensing for cancer biomarker: approaches and directions. *Bioact. Mater.*.

[48] Das G. M., Managò S., Mangini M., De Luca A. C. (2021). Biosensing using sers active gold nanostructures. *Nanomaterials*.

[49] Arabi M. (2021). Label-free SERS detection of Raman-Inactive protein biomarkers by Raman reporter indicator: toward ultrasensitivity and universality. *Biosens. Bioelectron.*.

[50] Zhou L. (2023). The label-free detection and identification of SARS-CoV-2 using surface-enhanced Raman spectroscopy and principal component analysis. *Biosensors (Basel)*.

[51] Rippa M. (2021). SERS biosensor based on engineered 2D-aperiodic nanostructure for in-situ detection of viable brucella bacterium in complex matrix. *Nanomaterials*.

[52] Freedman S. B., van de Kar N. C. A. J., Tarr P. I. (2023). Shiga toxin–producing Escherichia coli and the hemolytic–uremic syndrome. *N. Engl. J. Med.*.

[53] Liu Y., Thaker H., Wang C., Xu Z., Dong M. (2023). Diagnosis and treatment for shiga toxin-producing Escherichia coli associated hemolytic uremic syndrome. *Toxins*.

[54] Melton-Celsa A. R. (2014). Shiga toxin (stx) classification, structure, and function. *Microbiol Spectr*.

[55] Food and Agriculture Organization of the United Nations World Health Organization, Assessment (JEMRA), Nutrition and Food Safety (NFS), Standards & Scientific Advice on Food Nutrition (SSA) (2018). *Shiga Toxin-Producing Escherichia coli (STEC) and Food : Attribution, Characterization, and Monitoring: Report*.

[56] Costa-Ribeiro A., Lamas A., Mora A., Prado M., Garrido-Maestu A. (2024). Comparative performance of two different detection chemistries of qPCR for their implementation in a same-day detection method to determine the presence of Shiga toxin–producing Escherichia coli in ready-to-eat salads. *LWT*.

[57] Nada H. G., El-Tahan A. S., El-Didamony G., Askora A. (2023). Detection of multidrug-resistant Shiga toxin-producing Escherichia coli in some food products and cattle faeces in Al-Sharkia, Egypt: one health menace. *BMC Microbiol.*.

[58] Wasiewska L. A., Juska V. B., Seymour I., Burgess C. M., Duffy G., O’Riordan A. (2023). Electrochemical nucleic acid-based sensors for detection of Escherichia coli and Shiga toxin-producing E. coli—review of the recent developments. *Compr. Rev.*.

[59] Wasiewska L. A., Diaz F. G., Shao H., Burgess C. M., Duffy G., O’Riordan A. (2022). Highly sensitive electrochemical sensor for the detection of Shiga toxin-producing E. coli (STEC) using interdigitated micro-electrodes selectively modified with a chitosan-gold nanocomposite. *Electrochim. Acta*.

[60] O’Riordan A., Wasiewska L., Diaz F. G., Teixeira S. R., Burgess C. M., Duffy G. (2022). Amplification-free, highly sensitive electrochemical DNA-based sensor for simultaneous detection of stx1 and stx2 genes of Shiga toxin-producing E. coli (STEC). *ChemRxiv*.

[61] Joseph A., Cointe A., Kurkdjian P. M., Rafat C., Hertig A. (2020). Shiga toxin-associated hemolytic uremic syndrome: a narrative review. *Toxins*.

[62] Brigotti M. (2019). The structure of the Shiga toxin 2a A-subunit dictates the interactions of the toxin with blood components. *Cell. Microbiol*..

[63] Ardissino G. (2020). Is Shigatoxin 1 protective for the development of Shigatoxin 2-related hemolytic uremic syndrome in children?. *Pediatr. Nephrol.*.

[64] Nagatsuka T. (2013). Localized surface plasmon resonance detection of biological toxins using cell surface oligosaccharides on glyco chips. *ACS Appl. Mater. Interfaces*.

[65] Yaghoubi F., Zeinoddini M., Shoushtari M. (2023). Detection of shiga-like toxin produced by E. coli O157:H7 based on the LSPR property of gold nanoparticles. *J. Appl. Biotechnol. Reports*.

[66] Zhang Q., Liu Y., Nie Y., Ma Q., Zhao B. (2019). Surface plasmon coupling electrochemiluminescence assay based on the use of AuNP@C3N4QD@mSiO2 for the determination of the Shiga toxin-producing Escherichia coli (STEC) gene. *Microchim. Acta*.

[67] Wang B., Park B., Chen J., He X. (2020). Rapid and label-free immunosensing of Shiga toxin subtypes with surface plasmon resonance imaging. *Toxins (Basel)*.

[68] Yang Y., Wasiewska L. A., Burgess C. M., Duffy G., Lovera P., O’Riordan A. (2022). Detection of stx2 from Shiga toxin-producing Escherichia coli (STEC) by a surface enhanced Raman spectroscopy (SERS) sensor using recycled silicon chips. *Sens Actuators B Chem*.

[69] Ren K. (2024). A colorimetric and SERS dual-mode aptasensor for the detection of Shiga toxin type II based on Mn/Fe-MIL(53)@AuNSs. *Talanta*.

[70] Rippa M. (2022). Plasmonic metasurfaces for specific SERS detection of shiga toxins. *ACS Appl. Mater. Interfaces*.

[71] Palermo G. (2024). Intrinsic superchirality in planar plasmonic metasurfaces. *Nano Lett*..

[72] Rippa M., Castagna R., Kolkowski R., Zyss J., Zhou J., Petti L. (2020). Novel supra-molecular arrangements with plasmonic functionalities for fipronil pesticide detection. *SPIE-Intl Soc Optical Eng*.

[73] Matussek A. (2003). Molecular and functional analysis of Shiga toxin-induced response patterns in human vascular endothelial cells. *Blood*.

[74] Kudelski A. (2005). Characterization of thiolate-based mono- and bilayers by vibrational spectroscopy: a review. *Vib. Spectrosc*..

[75] Keskin S., Efeoğlu E., Keçeci K., Çulha M. (2013). Label-free detection of proteins in ternary mixtures using surface-enhanced Raman scattering and protein melting profiles. *J. Biomed. Opt.*.

[76] He P. (2023). Rapid and stable detection of three main mycotoxins in rice using SERS optimized AgNPs@K30 coupled multivariate calibration. *Food Chem.*.

[77] Podstawka E., Ozaki Y., Proniewicz L. M. (2004). Part I: surface-enhanced Raman spectroscopy investigation of amino acids and their homodipeptides adsorbed on colloidal silver. *Appl. Spectrosc*..

[78] Huang R., Ripstein Z. A., Rubinstein J. L., Kay L. E. (2020). Probing cooperativity of N-terminal domain orientations in the p97 molecular machine: synergy between NMR spectroscopy and cryo-EM. *Angew. Chem. Int. Ed.*.

[79] Podstawka E., Ozaki Y., Proniewicz L. M. (2004). Adsorption of S-S containing proteins on a colloidal silver surface studied by surface-enhanced Raman spectroscopy. *Appl. Spectrosc.*.

[80] Szekeres G. P., Kneipp J. (2019). SERS probing of proteins in gold nanoparticle agglomerates. *Front. Chem.*.

[81] Willets K. A. (2009). Surface-enhanced Raman scattering (SERS) for probing internal cellular structure and dynamics. *Anal. Bioanal. Chem.*.

[82] Fazio B. (2016). SERS detection of biomolecules at physiological pH via aggregation of gold nanorods mediated by optical forces and plasmonic heating. *Sci. Rep.*.

[83] Fang Y. (2021). Rapid and label-free identification of different cancer types based on surface-enhanced Raman scattering profiles and multivariate statistical analysis. *J. Cell. Biochem*..

[84] Bazylewski P., Divigalpitiya R., Fanchini G. (2017). In situ Raman spectroscopy distinguishes between reversible and irreversible thiol modifications in l-cysteine. *RSC Adv*..

[85] Lin V. J. C., Koenig J. L. (1976). Raman studies of bovine serum albumin. *Biopolymers*.

[86] Benevides J. M., Overman S. A., Thomas G. J. (2003). Raman spectroscopy of proteins. *Curr. Protoc. Protein Sci.*.

[87] Mocák J., Janiga I., Rábarová E. (2009). Evaluation of IUPAC limit of detection and ISO minimum detectable value-electrochemical determination of lead. *Nova Biotechnologica*.

[88] Silva C. J., Lee B. G., Yambao J. C., Erickson-Beltran M. L., Quiñones B. (2018). Using nanospray liquid chromatography and mass spectrometry to quantitate shiga toxin production in environmental Escherichia coli recovered from a major produce production region in California. *J. Agric. Food Chem.*.

[89] Beutin L., Zimmermann S., Gleier K. (2002). Evaluation of the VTEC-Screen “Seiken” test for detection of different types of Shiga toxin (verotoxin)-producing Escherichia coli (STEC) in human stool samples. *Diagn. Microbiol. Infect. Dis.*.

[90] Chien Y. Y. (2008). Globotriose-functionalized gold nanoparticles as multivalent probes for Shiga-like toxin. *Chembiochem.*.

[91] Kuo F. Y., Chang B. Y., Wu C. Y., Mong K. K., Chen Y. C. (2015). Magnetic nanoparticle-based platform for characterization of Shiga-like toxin 1 from complex samples. *Anal. Chem.*.

[92] To C. Z., Bhunia A. K. (2019). Three dimensional Vero cell-platform for rapid and sensitive screening of Shiga-toxin producing Escherichia coli. *Front. Microbiol.*.

[93] Lu T. (2019). Rapid detection of Shiga toxin type II using lateral flow immunochromatography test strips of colorimetry and fluorimetry. *Analyst*.

[94] Rippa M. (2022). Plasmonic metasurfaces for specific SERS detection of Shiga toxins. *ACS Appl. Mater. Interfaces*.

[95] Armstrong C. M., Ruth L. E., Capobianco J. A., Strobaugh T. P., Rubio F. M., Gehring A. G. (2018). Detection of shiga toxin 2 produced by Escherichia coli in foods using a novel AlphaLISA. *Toxins*.

[96] Ardissino G. (2020). Is Shigatoxin 1 protective for the development of Shigatoxin 2-related hemolytic uremic syndrome in children? Data from the ItalKid-HUS Network. *Pediatr. Nephrol.*.

[97] Brigotti M. (2020). Particulate shiga toxin 2 in blood is associated to the development of hemolytic uremic syndrome in children. *Thromb. Haemost.*.

