# Supplementary material for: Diagnostic oriented discrimination of different Shiga toxins via PCA-assisted SERS-based plasmonic metasurface
Source: Nanophotonics. 2025 Apr 17;14(23):4005–18. doi: 10.1515/nanoph-2024-0696 (PMC12617738; doi:10.1515/nanoph-2024-0696)
Supplement: Supplementary file 1 — Supplementary Material Details [file j_nanoph-2024-0696_suppl_001.docx]

Supplementary Material

**Diagnostic oriented discrimination of different shiga toxins via PCA-assisted SERS-based Plasmonic Metasurface**

M. Rippa^1*^, A. Milano^1,⊥^, V. Marchesano^1,⊥^, D. Sagnelli^1^, B. Guilcapi^1^, A. D’Avino^1^, G. Palermo^2,3^, G. Strangi^2,3,4*^, L. Consagra^5^, M. Brigotti^5,1^, S. Morabito^6,1^, J. Zyss^7^ and L. Petti^1^

^1^Institute of Applied Sciences and Intelligent Systems "E. Caianiello" CNR, Pozzuoli, Italy

^2^Department of Physics, NLHT-Lab, University of Calabria

^3^CNR-NANOTEC, Institute of Nanotechnology, 87036 Rende, Italy;

^4^Department of Physics, Case Western Reserve University, Cleveland, Ohio 44106, United States;

^5^Dipartimento di Scienze Mediche e Chirurgiche, Sede di Patologia Generale, Università di Bologna, Bologna, Italy

^6^Department of Food Safety, Nutrition and Veterinary Public Health, Istituto Superiore di Sanitá, Rome, Italy

^7^Lumière, Matière et Interfaces (LUMIN) Laboratory, Institut d’Alembert, Ecole Normale Supérieure Paris-Saclay, Université Paris Saclay, Gif sur Yvette, France

*Corresponding authors, E-mail: [m.rippa@isasi.cnr.it](mailto:m.rippa@isasi.cnr.it); [gxs284@case.edu](mailto:gxs284@case.edu); [maurizio.brigotti@unibo.it](mailto:maurizio.brigotti@unibo.it)

^⊥^The authors contributed equally to the work

**Near field enhancement computational modeling**

To calculate the enhancement of the near field (**E**/**E**_0_ – electric and **B**/**B_0_** - magnetic) of the nanostructures a Finite Element Method model in Comsol Multiphysics has been developed.

A 3D geometry consisting of a parallelepiped with the height depending on the specific wavelength considered in the calculation is modelled. The parallelepiped is constituted by 3 regions: starting from the bottom: i) the glass with a refractive index n_glass_ = 1.52; ii) the gold nanostructures (Johnson and Christy refractive index), and iii) the surrounding medium of the nanostructures constitute of air (n_air_= 1.0). To create a wave diffusion environment, an input and an output port were used, to simulate the incidence from the *−z* direction (top). To ensure the infinity conditions in the (x, y) plane, the Periodic Boundary Conditions (PBCs) were applied to the surfaces along the (x, y) directions of the blocks. To simulate the repeated single unit cell, the Floaquet periodicity was used. In addition to the PBCs, Perfect Matched Layers (PMLs) were introduced into the simulated system, to reproduce an absorbing boundary condition at the block extremes. The equations were solved by discretizing the problem, creating a dense mesh with control over the single components of the constructed geometry. In particular, for this study, an “extremely-fine” mesh was chosen.

Further details on the numerical model can be found in the authors' previous work. Ref [70] of the main work.

The calculated enhancement in the near-fields confirms that the structure characterized by the mid parameter of 100 nm has more significant values (|**E|**/**E**_0 MAX_ ≈ 162) than the structures having mid values between 25 - 150 nm.


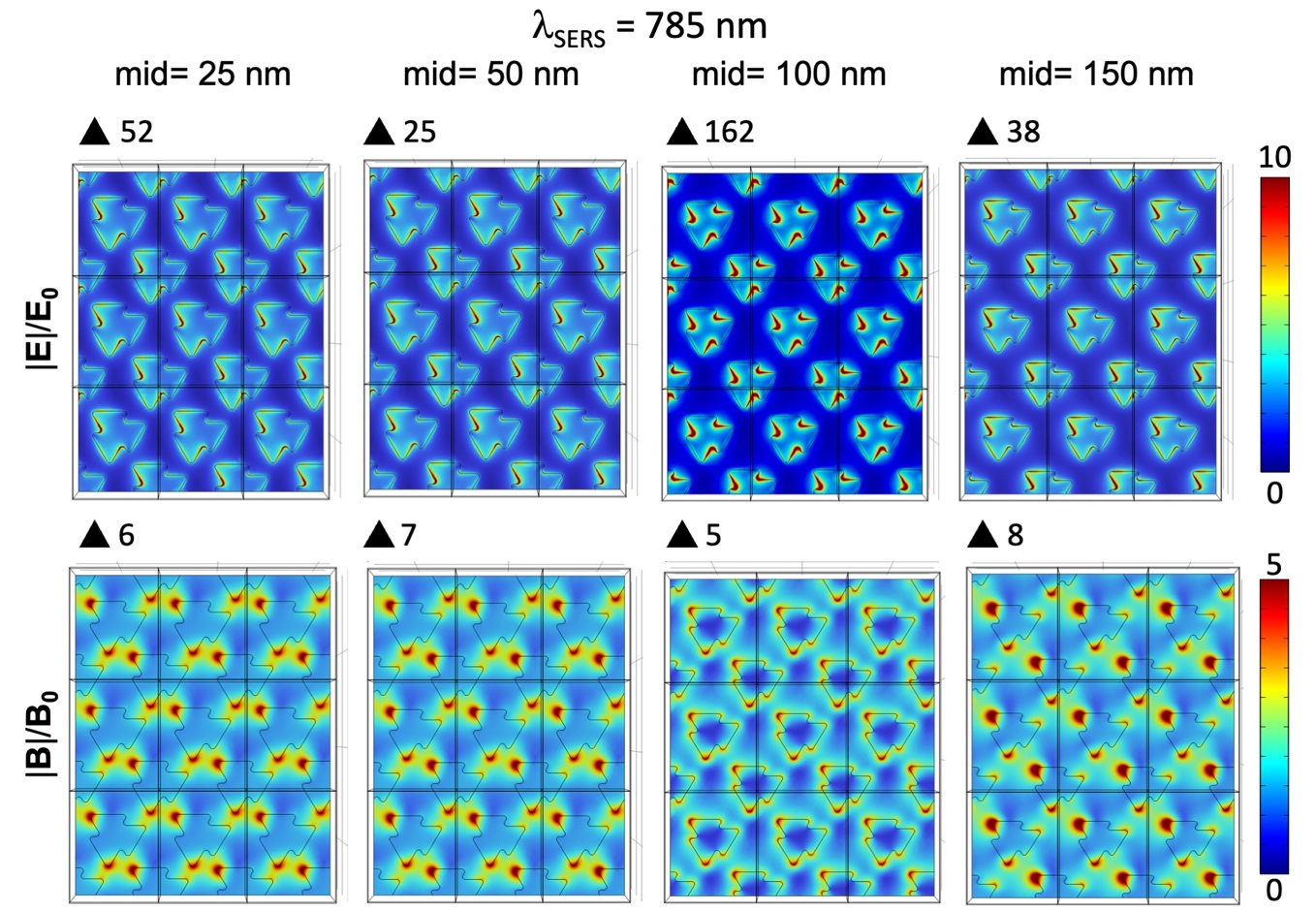


**Figure S1.** Normalized electric and magnetic field amplitude distributions calculate in the z=0 plane (air-gold interface) at the excitation wavelength of 785 nm for different mid parameter.

**Evaluation of Photoinduced Heating of the Substrate During SERS Measurement**

In order to assess the temperature variation of the sample under the laser radiation exposure, a photothermal measurement was conducted. As reported in the main article, the SERS measurement was performed using a 785 nm laser source with a beam power of approximately 12 mW, focusing the beam through a 50X objective with a N.A. of 0.75. The laser beam intensity was approximately 1.7 10^3^ W/cm². Using a high-resolution thermal camera, (FLIR A655sc) equipped with a close-up IR lens providing a 2.9X magnification factor and a spatial resolution of 50 µm at a reduced working distance—the photothermal effect induced by the laser radiation on the sample was measured. This measurement allowed for precise mapping of the heat distribution and temperature gradients across the sample.

As observed from the acquired photothermal image, the intensity used for the SERS measurements does not lead to a temperature increase in the sample.”


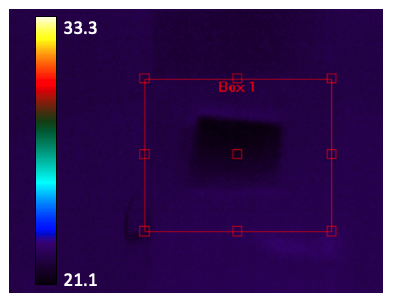


**Figure S2**. Photothermal image of the sample illuminated with the same intensity used in the SERS measurements.

**Fingerprint analysis**

The spectral analysis was initiated from the raw data, followed by baseline correction to mitigate background interference. The baseline was computed using the asymmetric least squares smoothing algorithm, optimizing for minimal distortion of the spectral features. After this, the spectra were normalized between 0 and 1, employing the standard transformation

$$\frac{(I-I_{min})}{(I_{max}-I_{min})}$$

​​here *I* denotes the intensity values. Peak identification was performed by calculating the first derivative of the spectra, with a 5% threshold applied to capture significant spectral features. Finally, the spectra were stacked to facilitate comparative analysis and highlight the peak positions across samples.

**Baseline correction**

The baseline correction is a critical step in spectral analysis because it helps to remove the background signal or drift that can obscure the true features of the spectra, such as peaks. In Origin pro there are various ways to perform such endeavor but the one used here for SERS spectra is the asymmetric least squares smoothing algorithm. This algorithm is very robust for non-uniform baselines (our case) and adaptable.

**Normalization and Peak detection**

At this stage normalization is advised to facilitate the peak detection or fitting to find areas etc. Sometime a smoothing is advised too but, in this case, non-necessary. Peak detection via the first derivative method is based on identifying zero-crossings in the first derivative of a spectrum. Adding a threshold usually 20% we remove the noise, in this case 5% was used to avoid losing some peaks. But we could just focus on the peaks above 20%. Subsequently this processing the spectra were stacked to check out the peaks in common Figure 2. The peaks are reported in a table and assigned.

**Vibration modes**

**Table S1.** SERS peak positions and tentative vibrational mode assignments

| **Wavenumber (cm⁻¹)** | **Assignation** | **Stx1a** | **Stx2a** | **Stx2a (cl)** |
| --- | --- | --- | --- | --- |
| 470 | nd | + | + | - |
| 489 | nd | + | + | + |
| 514-544 | S-S stretching [69] | + | + | + |
| 586 | nd | + | + | + |
| 624 | n(C–S)G [90]  Phe [91] | + | + | + |
| 657 | C-H in-plane bending,  Stretch of H39 on C16, strong swagging of H42 on C24 and H28 on N2, H37 on O12, out of plane breathing of benzene ring [75] | + | + | - |
| 671 | Isoleucine/C-S stretching [69,76] | - | - | + |
| 707 | Tryptophan / C-S stretching [91] | + | + | + |
| 720 | Ile [77]  met C–S stretching [69]  n(C–S)T [73]  Tryptophan [78] | + | + | - |
| 731 | Tryptophan [78] | - | - | + |
| 778 | Tryptophan ring breathing [78] | + | + | + |
| 825 | Tyrosine ring breathing mode [85, 92] | + | + | + |
| 836 | backbone stretching/out of plane ring breathing in tyrosine [69] | + | + | + |
| 869 | Tyrosine [91] | + | + | + |
| 891 | Tryptophan/ C-C stretching [78] |  |  | + |
| 935 | Tryptophan/ C-C stretching [78]  δ(C-C-N)symm , α-helical skeletal [79, 80] | + | + | + |
| 1001 | Symmetric ring breathing mode of phenylalanine [81]  Symmetric CC ring stretching [81] | + | + | + |
| 1029 | C–H stretching mode of phenylalanine [82] | + | + | + |
| 1067 | C-N stretching [78] | + | + | + |
| 1087 | nd (PBS o C-N) [81] | + | + | + |
| 1139 | C-N /C-H stretching [76] | + | + | + |
| 1166 | C-C stretching in proteins [81] | + | + | + |
| 1188 | Cyt/Gua/Ade [81] | + | + | + |
| 1216 | Amide III band [81] | + | + | + |
| 1252 | Amide III band -N–H in-plane bend, C–N stretch [78], [80] | + | + | + |
| 1279 | Amide III band [78]  CH2 wag or Ring stretching [81] | + | + | + |
| 1323 | Trp/C-H deformation/Backbone/C-N twisting [76]  C–H stretching of adenine [82] | + | + | + |
| 1381 | COO- stretching [78] | + | + | + |
| 1439 | C-H2/C-H3 deformation [81] | + | + | + |
| 1491 | Probably C-H2/C-H3 deformation [81] | + | + | + |
| 1513 | His and/or Trp [78] | + | + | + |
| 1552 | COO^-^stretching /His/ Trp /Phe [69] | - | - | + |
| 1574 | Amide II band - Ade/Gua [78] | + | + | + |
| 1626 | Tyr/Trp/Phe [78] | + | + | + |
| 1684 | Amid I band [78] | + | + | + |
| 1709 | nd |  |  |  |
| 1723-1836 | C=O stretch and C-C stretch [83, 84] | + | - | - |
| 1844 - 2444 | nd | + | + | + |
| 2510 | S-H stretching, thiol group exposure [85] | - | - | + |
| 2524 | S-H stretching, thiol group exposure [85] | + | + | - |
| 2535 | S-H stretching, thiol group exposure [85] | + | + | - |

References of the table (not present in the main manuscript)

90. A.A. Mankova, A.I. Nagaeva, N.N. Brandt, A.Yu. Chikishev. Cleavage of disulfide bonds used to reveal manifestation of tertiary structure in the Raman spectra of proteins.Vibrational Spectroscopy, 128, 2023, 103564.

91. Chih-Hsien Wang, Chia-Chi Huang, Long-Liu Lin, Wenlung Chen. The effect of disulfide bonds on protein folding, unfolding, and misfolding investigated by FT–Raman spectroscopy. Journal of Raman Spectroscopy, 47, 8, 2016, 940-947.

92. A. Rygula, K. Majzner, K. M. Marzec, A. Kaczor, M. Pilarczyk, M. Baranska. Raman spectroscopy of proteins: a review. J. of Raman Spectroscopy, 44, 8, 2013, 1061-1076.

**ANOVA analysis**

- **Unfunctionalized SERS measurements**

To validate the ability of the PCA model to differentiate between the three toxin groups, an analysis of variance (ANOVA) was conducted on the first two principal components. For PC1, the ANOVA revealed a highly significant group effect (F^2,117^=531, p< 2×10^−16^), indicating that this component robustly discriminates among the toxin groups. Similarly, PC2 also demonstrated a significant group effect (F^2,117^=775.4, p< 2×10^−16^), further highlighting its strong contribution to group separation. These results unequivocally confirm the effectiveness of the PCA model in capturing the variance associated with the toxin groups. The exceptionally low p-values and high F-statistics for both components underscore the robustness of this approach, establishing PCA as a powerful and reliable tool for toxin discrimination in complex datasets.

PC1

Df Sum Sq Mean Sq F value Pr(>F)

Group 2 0.005640 2.82e-03 531 <2e-16 ***

Residuals 117 0.000621 5.30e-06

PC2

Df Sum Sq Mean Sq F value Pr(>F)

Group 2 0.003215 0.0016077 775.4 <2e-16 ***

Residuals 117 0.000243 0.0000021

- **Functionalized SERS measurements**

Building upon the initial analysis, where PCA was validated as a powerful tool for differentiating toxin groups based on their spectral data, a similar approach was applied to evaluate the discrimination achieved using antibody-based detection. ANOVA on the first two principal components confirmed the remarkable ability of the PCA model to distinguish the three toxin groups. For PC1, a highly significant group effect was observed (F^2,95^=881.5, p < 2×10^−16^), demonstrating that this component captures the major variance related to toxin group differentiation. Likewise, PC2 also exhibited a strong and significant group effect (F^2,95^ = 501.4, p < 2×10^−16^), further supporting the discriminative power of the model. These findings reinforce the robustness of PCA across different experimental conditions and detection methods, confirming its reliability and adaptability for accurately separating toxin groups in complex analytical scenarios.

PC1

Df Sum Sq Mean Sq F value Pr(>F)

Group 2 0.005036 0.0025181 881.5 <2e-16 ***

Residuals 95 0.000271 0.0000029

PC2

Df Sum Sq Mean Sq F value Pr(>F)

Group 2 0.003401 1.7e-03 501.4 <2e-16 ***

Residuals 95 0.000322 3.4e-06
